# Response Time as an Implicit Self-Schema Indicator for Depression Among Undergraduate Students: Preliminary Findings From a Mobile App–Based Depression Assessment

**DOI:** 10.2196/14657

**Published:** 2019-09-13

**Authors:** Kyungmi Chung, Jin Young Park, DaYoung Joung, Kyungun Jhung

**Affiliations:** 1 Department of Psychiatry Yonsei University College of Medicine Gangnam Severance Hospital, Yonsei University Health System Seoul Republic of Korea; 2 Institute of Behavioral Science in Medicine Yonsei University College of Medicine Seoul Republic of Korea; 3 Department of Psychiatry International St. Mary’s Hospital Catholic Kwandong University College of Medicine Incheon Republic of Korea

**Keywords:** depressive symptoms, response time, self-concept, mobile phone, mobile apps, diagnostic screening programs, self-assessment, treatment adherence, compliance

## Abstract

**Background:**

Response times to depressive symptom items in a mobile-based depression screening instrument has potential as an implicit self-schema indicator for depression but has yet to be determined; the instrument was designed to readily record depressive symptoms experienced on a daily basis. In this study, the well-validated Korean version of the Center for Epidemiologic Studies Depression Scale-Revised (K-CESD-R) was adopted.

**Objective:**

The purpose of this study was to investigate the relationship between depression severity (ie, explicit measure: total K-CESD-R Mobile scores) and the latent trait of interest in schematic self-referent processing of depressive symptom items (ie, implicit measure: response times to items in the K-CESD-R Mobile scale). The purpose was to investigate this relationship among undergraduate students who had never been diagnosed with, but were at risk for, major depressive disorder (MDD) or comorbid MDD with other neurological or psychiatric disorders.

**Methods:**

A total of 70 participants—36 males (51%) and 34 females (49%)—aged 19-29 years (mean 22.66, SD 2.11), were asked to complete both mobile and standard K-CESD-R assessments via their own mobile phones. The mobile K-CESD-R sessions (binary scale: yes or no) were administered on a daily basis for 2 weeks. The standard K-CESD-R assessment (5-point scale) was administered on the final day of the 2-week study period; the assessment was delivered via text message, including a link to the survey, directly to participants’ mobile phones.

**Results:**

A total of 5 participants were excluded from data analysis. The result of polynomial regression analysis showed that the relationship between total K-CESD-R Mobile scores and the reaction times to the depressive symptom items was better explained by a quadratic trend—F (2, 62)=21.16, *P*<.001, *R*^2^=.41—than by a linear trend—F (1, 63)=25.43, *P*<.001, *R*^2^=.29. It was further revealed that the K-CESD-R Mobile app had excellent internal consistency (Cronbach alpha=.94); at least moderate concurrent validity with other depression scales, such as the Korean version of the Quick Inventory for Depressive Symptomatology-Self Report (ρ=.38, *P*=.002) and the Patient Health Questionnaire-9 (ρ=.48, *P*<.001); a high adherence rate for all participants (65/70, 93%); and a high follow-up rate for 10 participants whose mobile or standard K-CESD-R score was 13 or greater (8/10, 80%).

**Conclusions:**

As hypothesized, based on a self-schema model for depression that represented both item and person characteristics, the inverted U-shaped relationship between the explicit and implicit self-schema measures for depression showed the potential of an organizational breakdown; this also showed the potential for a subsequent return to efficient processing of schema-consistent information along a continuum, ranging from nondepression through mild depression to severe depression. Further, it is expected that the updated K-CESD-R Mobile app can play an important role in encouraging people at risk for depression to seek professional follow-up for mental health care.

## Introduction

### Background

Why do most psychometric instruments screen for or diagnose mental health problems (eg, depression, anxiety, and stress) only based on a summed total score, requiring that the same items be administered to all individuals? On the grounds of classical test theory [1-3], traditional psychometric measurements for depressive symptoms, such as the Beck Depression Inventory-II [4], the Patient Health Questionnaire-9 (PHQ-9) [5], the Geriatric Depression Scale [6], and the Center for Epidemiologic Studies Depression Scale-Revised (CESD-R) [7], have assumed that all items are equally weighted and that the characteristics of items cannot be separated from those of the person. In an attempt to reduce the burden on respondents of repeated exposures to long, fixed questionnaires, a number of researchers have, until recently, developed and validated a short-form of the self-report depression screening scales [8-10]. Given that there is the trade-off between efficiency and accuracy, a brief, efficient instrument can apply the fewest items to all respondents but may fail to employ the most informative items required to adequately and accurately measure the full range of clinical severity for each respondent. On the contrary, item response theory (IRT) [11,12] has not assumed that all people are measured with the same level of certainty, in that individuals with the same total score may display a wide variation in the relative severity and frequency of depressive symptoms. To deal with these limitations of existing, classical test, theory-based instruments, computerized adaptive testing based on IRT has been widely adopted; this has been used to estimate the respondent’s true score on the latent trait of interest in the individual item, thereby ensuring that a small, optimal number of items are administered to each individual until a previously determined level of measurement precision of the severity estimate is obtained [13].

Previous studies on the development of IRT-based computerized adaptive testing for depression [14-17] have had a greater emphasis on increased efficiency without loss of accuracy in assessing the presence and severity of depressive symptoms. However, what these studies have neglected is that examining the potential existence and function of a self-schema in nondepressed and depressed individuals should come first. In a cognitive model of the self [18], the self is viewed as a schema whose content is built up and organized from the individual’s day-to-day experiences in his or her world. As an interpretive frame for the encoding of personal data, the self-schema is activated and becomes an important part of the information processing system when the individual encounters personally relevant information. In Beck’s cognitive model of depression, depressive or negative content is defined as “an enduring characteristic of the cognitive organization, present in the depression-prone individual, even when the person is not feeling depressed [19].” Due to the enduring nature of negative schemata that contribute to the occurrence and reoccurrence of other depressive symptoms [20], the existence of negative self-referent information in individuals with different levels of depression (ie, nondepressed, high-risk individuals) needs to be tracked in their everyday life.

According to a self-schema model for depression, self-referent recall enhancement can be achieved only when schema-consistent information is processed in a highly efficient manner via one’s view of self, particularly for nonclinical and clinical depressives [21,22]. With a well-organized and efficient cognitive schema, the two groups would exhibit shorter response times to recall negative or depressive content in *yes/no* ratings for the self-referent judgement on experienced depressive symptoms; however, they would represent the only substantial difference in the actual content of personal information schematically represented [23]. Nonclinical and clinical depressives with similar total scores in severity of depression would produce a *content-specific* depressive self-schema, showing changes in the self-reported frequency of experienced symptoms. The self-schema model postulates not only an organizational breakdown, but also a subsequent return to effective processing of schema-consistent information [23]. In terms of the content and efficiency parameters, it would be difficult for mild depressives with a disruption in their organized and consistent view of self to efficiently process either positive or negative personal information. The lack of efficiency may result from their uncertainty regarding applicable self-referent attributes, which, in turn, would exhibit longer response times for self-referent judgements on negative information. In other words, those who have already begun to experience depressive symptoms and view themselves with negative or depressive content in their self-schema may have difficulty in the positive and precise identification of symptom severity; this is the case because positive or nondepressive content has yet to be displaced [24]. In this respect, the self-schema of mild depressives whose depression level is not severe enough to use a negative self-schema would differ from that of nonclinical and clinical depressives.

In general, it has been assumed by most cognitive theories and related works [25-27] that implicit cognitive biases stemming from activated negative self-schemata would be evident across all study designs (ie, measurement paradigms) and facets of cognition (ie, attention, memory, self-belief and interpretation, and self-esteem). Not until triggered by environmental stress do individuals vulnerable to depression possess relatively stable, negative, self-referential implicit cognitions that remain latent [25,26]. Because of their latent trait, these cognitions are posited to affect all aspects of information processing when activated [28,29]. Particularly, automatic (ie, implicit) dysfunctional attitudes about the self, known as the key vulnerability factor for the first onset and recurrences of depression [25,30], are more likely to remain undetected if self-report questionnaires are administered to explicitly measure their beliefs and feelings. From a dual-process perspective [26], the autonomic (ie, reflexive) nature of implicit cognitions can be assessed by means of reaction time or memory association measures, such as the Implicit Associations Test [31], compared to explicit cognitions measured by individuals’ deliberate (ie, reflective) consideration. If it may be possible to identify depression-vulnerable individuals and their self-schema based on patterns of explicit and implicit cognitions, using a person-level approach will be recommended to investigate how each self-schema is uniquely associated with different levels of depression severity [27]. With advancements in hardware and software technology, a wide variety of computerized implicit measures could be run on mobile devices, such as mobile phones and tablets, as well as in a laboratory or other experimentally controlled setting. However, explicit and implicit measures based more on the standard approach may contribute to relatively low accessibility for self-administered depression assessment tools in one’s daily life.

For measuring an accurate *latent trait of interest* in schematic self-referent processing of depressive symptom items, a mobile-based experience sampling method, also known as mobile-based ecological momentary assessment (mEMA), can be utilized. As a *time-stamped*, self-reported data collection method [32-34], mHealth apps can help users capture momentary psychological symptoms in their everyday lives in a timely and unconscious manner by recording entry and completion times with high contextual precision. More importantly, the apps can motivate those at high risk for depression to seek professional help, to discuss their screening test results with mental health care practitioners and professionals, and to take appropriate action against previously undiagnosed mental health problems [33,35,36]. While a majority of researchers have recently developed and validated a mobile-based *prospective* assessment tool [37-39], others have chosen and improved one of the well-established, standardized screening or diagnostic instruments to be optimized for mobile platforms [33,40-42]. While many measures ask an individual to recall depressive symptoms present in the previous weeks, mEMA is less influenced by recall bias, as individuals report symptoms that were present on that day. Taken together, there is a need to bridge the gap between standard and applied depression assessment tools. A number of depression apps available in app stores featured a therapeutic treatment (33.7%) or psychoeducation (32.1%) function, followed by medical assessment (16.9%), symptom management (8.2%), and supportive resources (1.6%) [43]. However, it should be determined whether the use of mobile phone and app technology in screening and management of depression is ecologically and clinically valid in order for these technologies to be employed in clinical practice as well as in large-scale epidemiological studies.

### Objective

The objective of this study is to allow people to readily record depressive symptoms they have experienced on a daily basis via their own mobile phones. For this purpose, we chose the Korean version of the CESD-R (K-CESD-R) [44], which is available for public use as one of the most widely used and well-validated depression screening instruments in the field of psychiatric epidemiology. We developed the K-CESD-R Mobile app in our previous study [42]. The purpose of this study is to examine the relationship between depression severity (ie, explicit measure: total K-CESD-R Mobile scores) and the latent trait of interest in depressive symptom items (ie, implicit measure: response times) in undergraduate students who had never been diagnosed with, but were at risk for, major depressive disorder (MDD) or comorbid MDD with other neurological or psychiatric disorders. We could thereby trace and understand the possible differences in schematic self-referent processing along a continuum ranging from nondepression through increasing levels of severity to clinical depression. It can be hypothesized that participants would more quickly respond to schema-compatible information than schema-incompatible information, thus presenting an inverted-U pattern between the total scores and response times. Based on the findings of this study, the potential of response times to depressive symptom items as an implicit self-schema indicator for depression will be determined. Furthermore, methodological discussion will be helpful to enhance the quality of the depression assessment to be used in both community and clinical samples.

## Methods

### Recruitment

This study was part of a government-driven project for developing mobile app-based intervention technology to identify South Korean college and university students vulnerable to mental health problems and to help them seek professional help. Therefore, undergraduate students who were 19 years of age or older and had never been diagnosed with either MDD or comorbid MDD with other neurological or psychiatric disorders were eligible to participate in this study. The study application was posted via online advertisements on several university websites in Seoul, South Korea. The online advertisement included the following information: aim of the study, inclusion and exclusion criteria, reward for participation, number of target participants, study period and procedure, and contact information.

A total of 70 undergraduate students—36 males (51%) and 34 females (49%)—who returned the participation application via email were recruited as healthy controls; they ranged in age from 19 to 29 years (mean 22.66, SD 2.11). In addition to the other inclusion and exclusion criteria for recruitment, the volunteers were required to have their own mobile phones with a screen size of at least 4 inches diagonally to control for variables that might affect reaction times. Based on Fitts’s Law [45], the size of a target (eg, either a *yes* or *no* button) to tap and its distance from the user’s current position (ie, hand gestures and fingertip locations) within the user interface had to be carefully considered. After providing signed informed consent, all the volunteers were enrolled. On the basis of the exclusion criteria for data analysis, those who did not assess depressive symptoms for at least 7 days and complete both standard and mobile K-CESD-R assessments were excluded from the statistical analysis. All were paid KRW 30,000 for their participation. This study was approved by the Institutional Review Board of Gangnam Severance Hospital.

In this study, we attempted to determine whether the updated K-CESD-R Mobile app could motivate its users to adhere to the self-administered assessment for 2 weeks. We also sought to determine whether the app could motivate those at risk for depression to seek further diagnostic interviews, as provided by the guidance on the interpretation of test results from the K-CESD-R Mobile app. As we intended to observe the adherence rate by the users of the app, information on further follow-up after finishing the 2-week course of the standard and mobile K-CESD-R assessments was not given to volunteers via the advertisement.

### Standard K-CESD-R Scale Versus Applied K-CESD-R Mobile App

To overcome the limitation of the retrospective recall-based K-CESD-R assessment, we had previously developed *K-CESD-R Mobile*, a mobile-optimized, daily self-report, depression screening tool; for a review, see Chung et al’s study [42]. Based on a *frequency* approach, an original version of the K-CESD-R scale instructed participants to indicate how often they have experienced each of the 20 symptom items, as defined by the fourth edition of the Diagnostic and Statistical Manual of Mental Disorders (DSM-IV) criteria. Participants were to indicate the frequency of symptoms over the past 2 weeks using the following 5-point response format ranging from 0 to 4: 0 (*Not at all* or *less than one day*), 1 (*1-2 days*), 2 (*3-4 days*), 3 (*5-7 days*), and 4 (*Nearly every day for 2 weeks*) [7]. In addition, the K-CESD-R Mobile app asked participants to indicate whether or not they have experienced depressive symptoms (20 items) in the past 24 hours using a *Yes* or *No* response format for the following 2 weeks. Responses of *Yes* and *No* were coded as 1 and 0, respectively. It was recommended to them that each session should be completed 24 hours after the previous session. Otherwise, participants could freely complete the assessment at any time within the specific time window of 6 hours before or after the recommended time (ie, every 24 hours), as displayed on the home screen of the app.

Furthermore, the K-CESD-R Mobile app applied a *ratio* approach to deal with the problem of missing data in case participants administered the assessment for at least 7 days or more, but not for all the days, during the 2-week study period. If participants completed less than 7 daily sessions during the study period, their final scores were not computed after completing the final session. To apply the same standard to compare the two K-CESD-R scores, we developed a new algorithm to convert a binary response to a 5-point response with different cutoff criteria: 0 (0 ≤ Y < 2/14), 1 (2/14 ≤ Y < 5/14), 2 (5/14 ≤ Y < 9/14), 3 (9/14 ≤ Y < 13/14), and 4 (13/14 ≤ Y=14/14); Y=Q/P, where Q is equal to the total number of times users responded *Yes* to each item, and P is equal to the total number of days that users completed sessions over 2 weeks. We tested the feasibility and validity of the K-CESD-R Mobile scale and its converting algorithm in our previous research [42]. According to the validation study of the K-CESD-R [44], both of the total K-CESD-R scores could range from 0 to 80, with a cutoff score of 13 or more.

In addition to the K-CESD-R scores, response times were recorded as the latent trait of interest in schema-compatible information, particularly on depressive symptoms experienced. The *latent trait of interest* was defined as the interval between the initial presentation of each item via the K-CESD-R Mobile app installed on participants’ mobile phones and their *Yes* or *No* responses to the item. To minimize the Hawthorne effect and improve the generalizability of results, no records of the response times were displayed on the app, nor were participants informed that their response time data would be collected. Only authorized investigators were allowed to access the raw response time data by signing in to a Web dashboard with a user ID and password and unlocking the data file with a different password.

### Procedure

For the initial visit, participants gathered at the same time in the grand auditorium at Gangnam Severance Hospital. Participants who returned their signed informed consent forms were asked to fill out a paper-and-pencil prequestionnaire to collect their demographic information and self-report ratings on depression scales, such as the Korean version of Quick Inventory for Depressive Symptomatology-Self Report (KQIDS-SR) [46,47] and the Korean version of the PHQ-9 [48].

To collect mental health data only from enrolled participants via the K-CESD-R Mobile app, they had to be registered in advance as beta testers of the app for the study period. iOS users were guided to download the beta app, K-CESD-R Mobile, only through Apple’s TestFlight platform for beta testing; Android users could directly search for and download the K-CESD-R Mobile app on the Google Play store. The K-CESD-R Mobile app required the participants’ consent to collect and use their data for research purposes under Korea’s Personal Information Protection Act, particularly at the final step of the sign-up process; this was to further ensure security and privacy for sensitive personal information collected and transmitted via mobile devices to a cloud services platform (ie, Amazon Web Services). After reviewing and agreeing to all terms of use and a privacy statement, participants could create an account and start a new session.

To lead them to perform a 2-week K-CESD-R test in a comfortable but controlled manner, a warm-up screen with a *Start* button was sequentially followed by a guidance screen and 20 K-CESD-R item screens. The guidance screen was presented to instruct participants that they could start a test when they were mentally ready to administer it. At the last item screen, participants were asked to tap a *Save and Send* button to transfer their responses to the Amazon Web Services platform. On the first visit, and even on the guidance screen, the app did not let participants know that response time data was being acquired while responding to the K-CESD-R items; the reason for this was to control the quality of an implicit measure as a self-schema indicator as well as to prevent participants from misunderstanding the aim of the study. In an effort to ensure adequate measurement of response times during the test, the app did not allow users to sign in or start the test unless a stable Internet connection via Wi-Fi or cellular network could be guaranteed; this was to prevent results being affected by Internet quality.

Following the completion of the first mobile K-CESD-R session, all participants received guidance from experimenters on the given tasks: (1) the remaining mobile K-CESD-R sessions should be administered on a daily basis for 2 weeks and (2) a standard K-CESD-R assessment created with SurveyMonkey [49] should be administered on the final day of the 2-week study period; this survey was delivered via text message and included a link to the survey. Accordingly, both mobile and standard K-CESD-R assessments ended on the same day.

After scoring was completed, participants whose online K-CESD-R score or mobile K-CESD-R score was 13 or above were recommended to have clinician-administered diagnostic interviews. It was explained to all participants at the first visit that the CESD-R was designed as a quick and reliable self-administered screening tool for depression, regardless of the platforms on which they were provided. To make the diagnosis of clinical depression, an initial screening of participants with these instruments would need to be followed by clinical interviews based on their K-CESD-R scores. Follow-up visits took place at the outpatient clinic in the Department of Psychiatry, Gangnam Severance Hospital; each participant was individually scheduled to come at a convenient time in order to motivate him or her to discuss mental health problems with a medical doctor. During the 30-minute clinical interview, the following scales were administered: the original English version of the Clinical Global Impressions-Severity of Illness Scale (CGI-S) [50], the Korean version of the Montgomery-Asberg Depression Rating Scale (K-MADRS) [51,52], the Korean version of the Hamilton Anxiety Rating Scale (K-HAM-A) [53,54], the Korean version of the Hamilton Depression Rating Scale (K-HAM-D) [55,56], and the Korean version of the Mini-International Neuropsychiatric Interview (MINI) version 5.0.0. [57,58]. After the second interview, further follow-up was not required.

### Statistical Analysis

Statistical analyses were performed using PASW Statistics 18 software (SPSS Inc). Cronbach alpha was calculated to evaluate the internal consistency of the standard and mobile K-CESD-R scales. As nonparametric alternatives to the paired-samples *t* test and Pearson’s correlation test, a Wilcoxon signed-rank test was used to compare the difference between the standard and mobile K-CESD-R scores whose normality assumptions were not satisfied. Spearman’s correlation coefficient was then calculated to measure concurrent validity of the K-CESD-R Mobile scale with other depression screening scales, such as the standard K-CESD-R, the KQIDS-SR, and the PHQ-9. In order to determine whether the relationship between the explicit and implicit self-schema measures for depression would be better explained by a quadratic trend than by a linear trend, the polynomial regression analysis was conducted after the normal distributions of the variables were confirmed by a Kolmogorov-Smirnov test.

## Results

### Participant Characteristics

After ensuring that enrolled undergraduate students met our inclusion and exclusion criteria for the statistical analysis, 5 out of the 70 participants were excluded (93% adherence rate). This is because the K-CESD-R Mobile app was designed to calculate the test results only if its users assessed depressive symptoms for at least 7 days in the 2-week study period. Furthermore, it was also required that students complete the standard K-CESD-R assessment on the last day of the mobile K-CESD-R assessment, following the experimental protocol of this study. The detailed demographic information on all participants included in the data analysis is presented in Table 1.

**Table 1:** Participant demographic characteristics (N=65).

Participant characteristic				Value
Age (years), mean (SD)				22.63 (2.13)
**Age (years), n (%)**				
		19		2 (3)
		20-29		63 (97)
**Gender, n (%)**				
		Male		32 (49)
		Female		33 (51)
**Mobile operating system, n (%)**				
		Android		35 (54)
		Apple iOS		30 (46)
Marital status (single), n (%)				65 (100)
**Current smoking status, n (%)**				
		Smoker		5 (8)
		**Duration of smoking in years, n (%)**		
			3	2 (3)
			4	1 (2)
			6	2 (3)
		**Cigarettes smoked per day, n (%)**		
			4	1 (2)
			8	1 (2)
			10	1 (2)
			11	1 (2)
			13	1 (2)
		Nonsmoker		60 (92)
**Current alcohol drinking status, n (%)**				
		Drinker		45 (69)
		**Frequency of alcohol intake per week, n (%)**		
			Once	25 (39)
			Twice	12 (19)
			Three times	7 (11)
			Four times	1 (2)
		Nondrinker		20 (31)

### Depression Screening by Self-Reported Scales and Clinician Interview

A total of 65 participants completed all the standard K-CESD-R assessments (median 3.00, interquartile range [IQR] 0-7.50; scored from 0 to 63) and the mobile K-CESD-R assessments (median 2.00, IQR 0-6.50; scored from 0 to 59) with high-variance distributions: coefficient of variation (CV) was 1.73 and 1.90, respectively. The distribution of the standard K-CESD-R scores had a positive skew (skewness 3.91, SE 0.30) and was leptokurtic (kurtosis 17.68, SE 0.59). Similarly, the mobile K-CESD-R scores had positively skewed (skewness 3.90, SE 0.30) and leptokurtic (kurtosis 17.39, SE 0.59) distributions. The internal consistencies of the standard K-CESD-R (Cronbach alpha=.94) and the mobile K-CESD-R (Cronbach alpha=.94) scales were equivalently high.

With use of a Wilcoxon signed-rank test, we found a significant difference between the standard K-CESD-R and mobile K-CESD-R scores (Z=–2.69, *P*=.007), with a Spearman’s correlation coefficient of .82 (*P*<.001). The number of participants whose depression screening score was 13 or above were as follows: (1) 9 participants based on the standard K-CESD-R, (2) 6 participants based on the mobile K-CESD-R, and (3) 5 participants based on both scales. Out of 10 participants, only 1 (10%) was consistently assessed as being above the diagnostic thresholds for depression through a clinician-administered diagnostic interview, structured using the CGI-S (score ≥3), the K-MADRS (score ≥16), the K-HAM-A (score ≥25), the K-HAM-D (score ≥19), and the Korean version of the MINI, with modules based on the DSM-IV diagnostic criteria for a major depressive episode. According to their standard and mobile K-CESD-R scores, all 10 of the participants at high risk for depression were advised to visit the clinic for a 30-minute clinical interview at their desired date and time. However, 2 out of 10 (20%) did not seek further professional follow-up, as recommended by both the K-CESD-R Mobile app and the experimenters.

### Concurrent Validity of the K-CESD-R Mobile Scale

The concurrent validity of the K-CESD-R Mobile scale was assessed through Spearman’s correlation between the K-CESD-R Mobile and other depression screening scales: KQIDS-SR with a total score of 0-26 (median 6.00, IQR 4.00-9.50, CV=.71; ρ=.38, *P*=.002) and the PHQ-9 with a total score of 0-19 (median 2.00, IQR 1.00-4.50, CV=1.11; ρ=.48, *P*<.001).

### Inverted U-Shaped Relationship Between Depression Score and Response Time

We tested the hypothesized curvilinear (ie, inverted U-shaped) relationship between the severity of participants’ depression level as an independent variable and personal negative information about themselves (ie, latent trait of interest) as a dependent variable; this was done whether the association between the two variables was best characterized by a quadratic trend or by a linear trend. The hypothesis was tested using a polynomial regression analysis. To justify the use of the parametric test, a Kolmogorov-Smirnov test was conducted for testing the normality assumption (Z=1.11, *P*=.17). Before hypothesis testing, we subtracted the mean response time for each item from each participant’s response time to control for the item effect only (ie, item length and vocabulary level). This is because the widely used double standardization method, which controlled for both person effects (ie, reading and motor speed) and item effects, was criticized for its artefactual negative correlation between items varying in mean response times [59]; it was also criticized because it was revealed that the severity of depressive symptoms accounted for impairments in information processing speed and psychomotor retardation [60].

**Figure 1:**
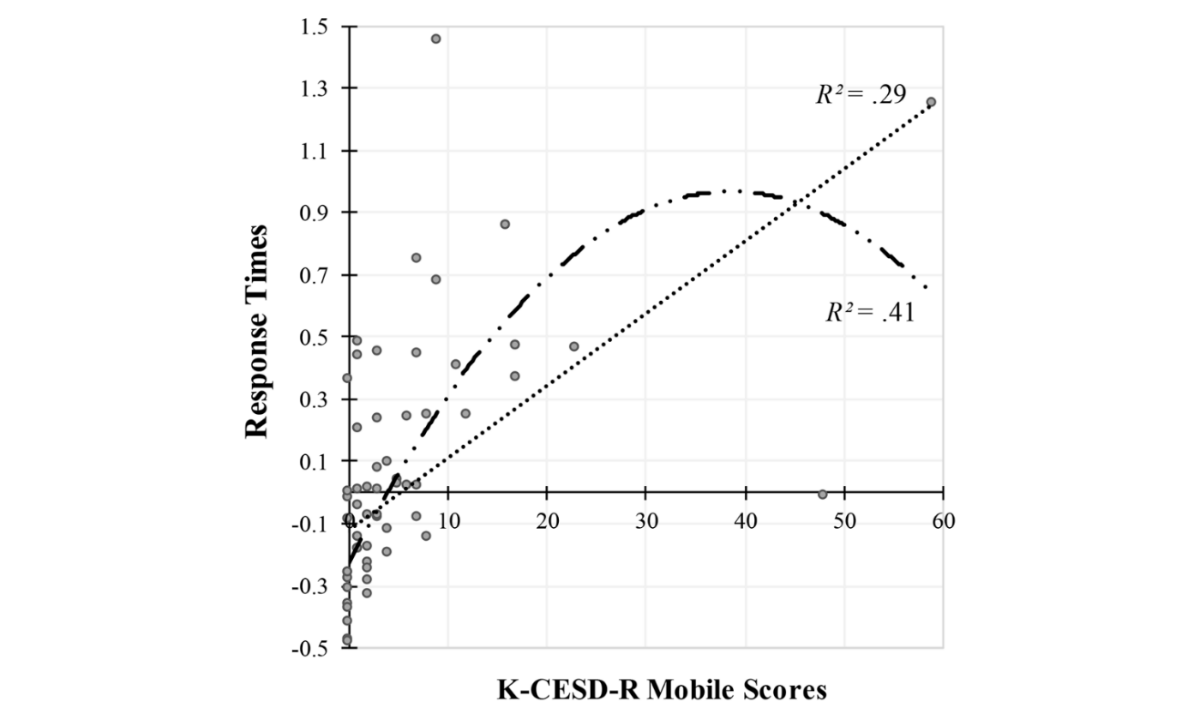
Results of polynomial regression analysis predicting the curvilinear relationship between total scores on the Korean version of the Center for Epidemiologic Studies Depression Scale-Revised (K-CESD-R) Mobile app and mean standardized reaction times for the depression items.

**Figure 2:**
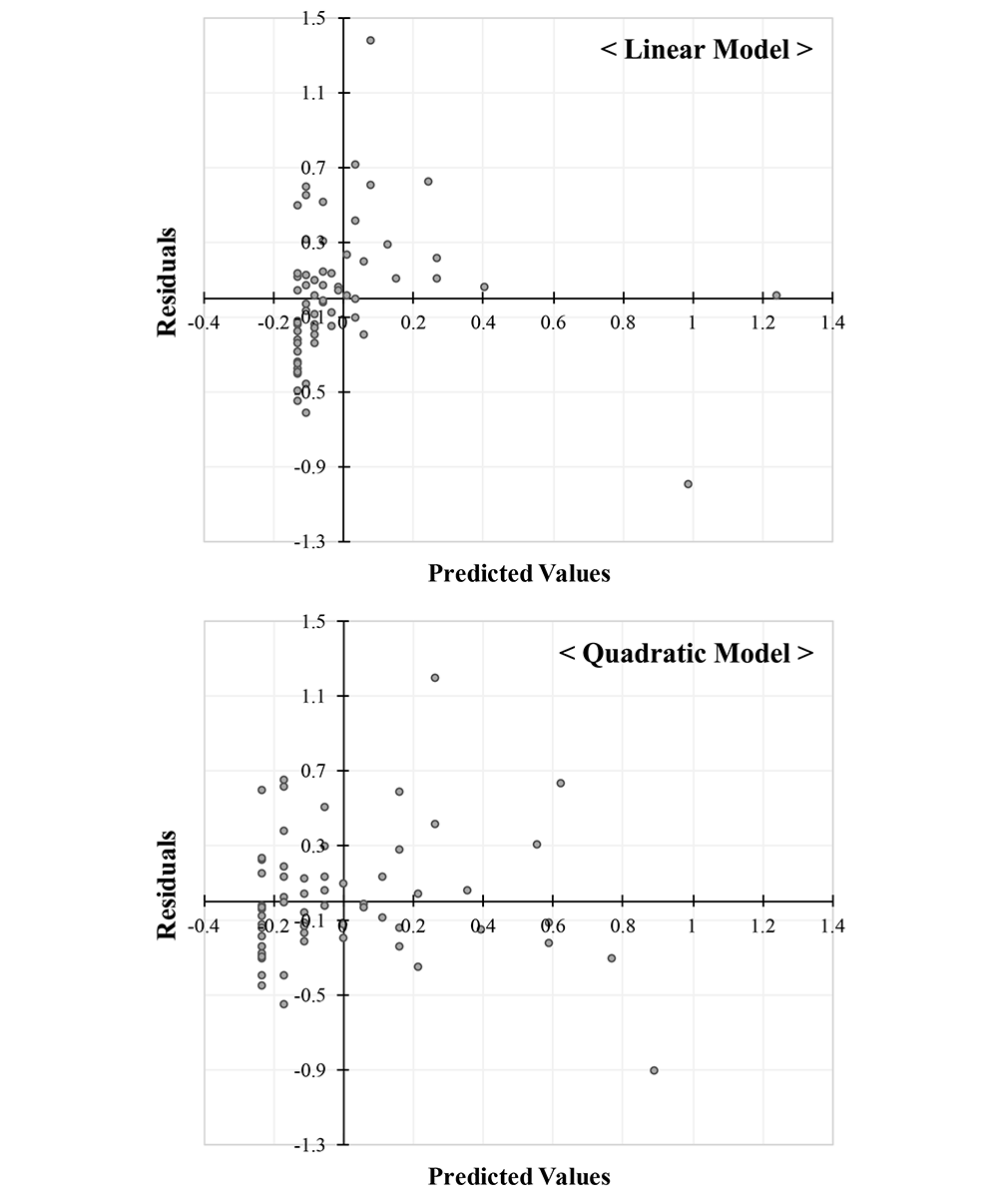
Scatterplots of residuals by fit values for linear and quadratic models.

The polynomial regression analysis revealed that the relationship between total K-CESD-R Mobile scores and the reaction times to the depressive symptom items was better captured by the quadratic trend—F (2, 62)=21.16, *P*<.001, *R*^2^=.41—than by the linear trend—F (1, 63)=25.43, *P*<.001, *R*^2^=.29. As shown in Figure 1, this finding reflects the inverted U-shaped reaction time effect as the self-schema evidence from faster reaction times for low and high K-CESD-R Mobile scores than for intermediate ones. To consider the quality of both models and the potential outliers, scatterplots of residuals by fit values for the linear model and quadratic model were produced. Figure 2 illustrates that the residuals of the quadratic model are more evenly dispersed than those of the linear model, showing their skewed distribution. It was also found that potential outliers are less identified in the quadratic model compared to that of the linear model.

## Discussion

### Principal Findings

The aim of this study was to investigate the relationship between the severity of depression and the latency of response to depressive symptom items in a sample of undergraduate students at risk for mental health problems. In this study, we postulated the potential existence and function of a depressive self-schema as the individual’s idiosyncratic cognitive structure, with which content (ie, item) and efficiency (ie, speed) would be responsive to variations in depression level when self-relevant information is processed. Given the impairment and breakdown process in the self-schema and subjective organization of personal information in depression, we hypothesized that nondepressed and severely depressed individuals would be faster than mildly depressed individuals in making self-referent judgments on experienced depressive symptoms with a *yes/no* response format, showing an inverted U-shaped pattern in their self-schemata.

As hypothesized by the self-schema model for depression [23], the inverted U-shaped relationship between the total K-CESD-R Mobile scores and response times to the items was found in a sample of young adult, university students who have not previously received a diagnosis of depression. It appeared to be empirically supported that individuals at risk of depression might exhibit a disruption in their organized and consistent views of self with both positive and negative information components; this is the case because positive information has yet to be displaced by negative or depressive information in their self-schema [23,24,61]. According to the statistical results and figures, the relationship between depression severity and reaction times was better explained by the quadratic model than the linear model. Figure 2 revealed that the residuals of the quadratic model were independent of the predicted fit values, and the residual distribution of the quadratic model was less skewed and more evenly dispersed than that of the linear model. It was observed in the quadratic model that potential outliers tended to be more acceptably scattered than in the linear model. However, our preliminary study had the limitations of sample size; as well, the study sample lacked participants who had been diagnosed with MDD as positive controls to show that the K-CESD-R Mobile app has the power and sensitivity to identify those students and demonstrate the inverted U-shaped relationship. In fact, we failed to determine whether the self-schema model for depression fit well with the inverted U-shaped curve in undergraduate students at all levels of depression. Beyond the limitations of the sample used and its size, it is also true that the response time, as an implicit self-schema indicator for depression, showed a *potential* for enhancing the quality of mobile app-based depression assessment and screening between nondepressed, mildly depressed, and severely depressed individuals.

Most of all, the K-CESD-R Mobile app was designed to make up for the weak points of traditional depression screening tools, which have rarely been used after the diagnosis of depression and have discriminated against individuals at risk for depression with optimal cutoff values. To develop a mobile-based depression intervention for identifying undergraduate students with mental health problems, we adopted the K-CESD-R Mobile app [42], with which individuals could assess their depressive symptoms experienced in the past 24 hours with *yes* or *no* ratings during a 2-week period. Considering that the standard K-CESD-R scale [44] asked respondents to choose response options from 1 to 5 based on how many days they have experienced the given symptoms during the past 1-2 weeks, the mobile K-CESD-R scale would contribute to reducing the possibility of recall bias from the retrospective depression assessment with a longer recall period. As far as the treatment for depression and the assessment of remission status are concerned, defining remission status from depression based only on the total scores of explicit symptom-based measures is not recommended; this is because there exists both discordance and concordance between the self-ratings of depression symptom severity and psychosocial functioning impairments [62]. As the next step, to estimate the respondents’ *true* scores, the app was updated to explicitly and implicitly measure depression severity on a daily basis by adding a new feature for acquiring the response latencies for all items with *total* K-CESD-R Mobile scores. The key feature was based on a developmental approach to the acquisition of *latent* negative self-schemata; prior depressive experience or repeated associations between current depressed mood and thoughts or memories is more likely to increase accessibility to negative cognitions once the self-schema has been activated [63]. Compared to the depression Implicit Associations Test that respondents should complete via computer- or mobile-based apps (eg, E-prime or Inquisit) in a controlled, uncomfortable environment to support the validity of the data collection, the K-CESD-R Mobile app installed on their own mobile phones automatically measures the response times to all items without requiring further test procedures. The mobile app employs a streamlined approach to a well-established depression screening tool for epidemiologic studies; therefore, the app would make it possible for both its users and practitioners to rapidly but accurately detect depressive symptoms and their severity and monitor any shift in the content and efficiency of the self-schema throughout the lifetime.

Moreover, the K-CESD-R Mobile app was expected to encourage positively screened individuals to seek professional help; this was to be done before an organizational breakdown and possible shifts for a subsequent return to efficient processing of schema-consistent information are completed. To do so, the K-CESD-R Mobile app provides clinicians and mental health professionals with access to the online dashboard to implement a personal-level intervention for individuals, simultaneously monitoring their adherence to the app and total scores from separate remote locations. However, we limited the scope of this study to the enhancement of the K-CESD-R Mobile app, not to that of its dashboard. Once participants produced their total scores on the last day of the standard and mobile K-CESD-R assessments, those whose online or mobile K-CESD-R scores (or both) were 13 or over (n=10) were directly contacted via text message and phone call, in order to make an appointment for the structured clinical interview. With an adherence rate of approximately 93% (65/70) on the depression assessments, 8 of the 10 participants (80%) visited the outpatient clinic for the diagnostic interview. In fact, it would be difficult to rule out the possibility that the high adherence rate to the app might result from the financial remuneration for their participation or the Hawthorne effect. Another possibility is that the participants perceived the app to be so credible that they decided to seek further medical attention for their depressive symptoms. Despite the benefits of successful screening and brief intervention for groups at high risk of depression, this study points out the need to further develop and implement more detailed, tailored, and evidence-based interventions for those whose depressive symptoms do not interfere with or cause difficulties in their lives. When they do not consider their psychosocial functioning as severely impaired, they are less likely to seek professional help and more likely to believe themselves to be in remission [62]. To shed light on this issue, we suggest major updates to enable users to decide what other explicit measures (eg, psychological impairment and quality of life) to include with the mobile K-CESD-R scale in the app. We also suggest that practitioners be allowed to send user-centered push notification messages via the online dashboard, thereby motivating users to adhere to the Internet- and mobile-based intervention platforms.

### Limitations

This study was part of a government-driven project for developing intervention technology to identify and help college and university students at high risk for mental health problems; therefore, the sample was only composed of undergraduate students from different university campuses who have not previously been diagnosed with clinical depression. Given the main purpose of this study to achieve the aim of the project, the sampling method limits the generalizability of the findings by recruiting students who are easily accessible and willing to participate in the research, in comparison to subclinical or clinical samples and even other nonclinical South Korean samples. Furthermore, the small sample size may lead this study to have insufficient power to identify clinically relevant differences. In addition to the sample size and characteristics, another concern about the rating algorithm used in the K-CESD-R Mobile app can be raised. To convert binary response data to 5-point response data, and to automatically calculate a total score if response data for at least 7 days was collected, we employed the ratio approach-based algorithm, which was developed in our previous study [42]. Despite this attempt to deal with the absence of response data from the possible missing days, the algorithm could be biased because the number of days that the mobile K-CESD-R scale had been completed could be influenced by the *yes* or *no* type of response. For example, people could be less likely to respond on the day when they were more depressed, which might underestimate their depression severity. Taken together, these limitations can be dealt with by replicating this experimental protocol and assessing test-retest reliability and validity of the app in a large sample of clinical depressives, as well as among nonclinical and subclinical depressives. This would allow us to test the feasibility of the intervention platforms and extend the findings of this study.

### Conclusions

In conclusion, this study showed preliminary evidence that the inverted U-shaped pattern of response times to all items would reflect the self-schema for depression, which was organized for the efficient processing of schema-consistent personal information on depressive symptoms experienced. High-risk adult students with unstable and incomplete depressive self-schemata, as well as mental health professionals, could benefit from measuring and analyzing response latency as an implicit self-schema indicator for depression; this could be done particularly via the K-CESD-R Mobile app and its compatible online dashboard for early intervention in depression management.

## Abbreviations

CESD-R: Center for Epidemiologic Studies Depression Scale-Revised
CGI-S: Clinical Global Impressions-Severity of Illness Scale
CV: coefficient of variation
DSM-IV: fourth edition of the Diagnostic and Statistical Manual of Mental Disorders
IQR: interquartile range
IRT: item response theory
K-CESD-R: Korean version of the CESD-R
K-HAM-A: Korean version of the Hamilton Anxiety Rating Scale
K-HAM-D: Korean version of the Hamilton Depression Rating Scale
K-MADRS: Korean version of the Montgomery-Asberg Depression Rating Scale
KQIDS-SR: Korean version of the Quick Inventory for Depressive Symptomatology-Self Report
MDD: major depressive disorder
mEMA: mobile-based ecological momentary assessment
MINI: Mini-International Neuropsychiatric Interview
PHQ-9: Patient Health Questionnaire-9
